# Diseases of the Kidney

**Published:** 1905-12-30

**Authors:** 


### DISEASES OF THE KIDNEY.

**Pathology of Bright's Disease.**—The modern tendency to ascribe disease to general rather than to local processes is shown in a recent discussion on nephritis at the American Medical Association. Thus Woods Hutchinson[1] believes that Bright's disease is not primarily a disease of the kidneys, but a toxæmia, in which the kidneys are damaged in the attempt to eliminate the toxin, and Councilman[2] insisted that nephritis was always a part of some infectious or toxic process. Stengel[3] showed that nephritis was frequently associated with various toxic conditions, but drew a sharp distinction between cases in which more or less inflammation of the kidney was found post-mortem or could be detected clinically and true cases of Bright's disease when the kidneys were extensively damaged and the symptoms mainly referable to this cause. Out of 581 cases which showed post-mortem evidence of nephritis only 51 were clinically acute or chronic Bright's disease, excluding interstitial nephritis. The latter condition or arteriosclerosis was present in 74 cases. The rest were cases of acute infection or cardiac disease, etc., and in many of these there was not even albuminuria during life. In the ætiology of nephritis attention has recently been called by Stalker[4] to tuberculosis and syphilis and chronic septic poisoning. He appears to think tubercle a potent predisposing cause, and though this view has been traversed it derives some support from the fact that at the Phipps Institute Fancine[5] states that in 53 cases of pulmonary tubercle only one kidney was found to be normal. Here again, however, the distinction pointed out by Stengel between clinical Bright's disease and pathological nephritis must be borne in mind.

**Salt Retention.**—Much interest at present centres in the question as to the relationship between sodium chloride retention and certain symptoms of Bright's disease. Dixon Mann,[6] in a paper on the causes and treatment of œdema, has summarised much of the recent literature on this subject. The theory put forward by those who hold that an excess of sodium chloride is the cause of the dropsy in renal disease is that the damaged epithelial cells being unable to excrete more than a certain amount of the salt, it is stored in the tissues, which consequently by osmosis abstract water from the blood vessels, this constituting œdema. In health, on the other hand, the kidneys can excrete any excess of sodium chloride, so that no dropsy occurs. Analyses of the tissues and fluids have not, however, given absolutely identical results in different cases, so that some consider that retention of water is the first effect of nephritis, and that salt retention is merely secondary to this. Richter,[7] for instance, found that in rabbits whose kidneys had been damaged by uranium nitrate the addition of salt to the ingested water made no difference in the amount of ascites. Others, again, consider that the retention of sodium chloride is not dependent on renal disease, but on some altered conditions in the tissues generally. There is, however, a very general consensus of opinion that some sort of relationship exists between the amount of sodium chloride in the body and the amount of œdema, and many observers report that good results follow when salt is excluded from the diet. Dixon Mann, in the paper referred to, states that although in many cases he could trace no constant relation between the amount of salt retained and the amount of œdema, in others they seemed to vary concomitantly. He therefore advises restriction in the intake of salt in Bright's disease. Kelly and Fife[8] have reported three cases of chronic nephritis in which the elimination of salt from the diet was followed by good results; they also consider that the amount of salt retained is an index of the severity of the renal lesion. Miller [9] states that in moderately severe cases sodium chloride may produce symptoms resembling uræmia, and Stengel [10] has described a case of chronic nephritis with acute attacks which occurred in a man who consumed large quantities of salt. When the salt was withheld great improvement in the patient's condition was noticed. Kövesi and Schulz,[11] who upheld the view that salt retention is the primary cause of renal dropsy, advise that not more than 8 grains of salt should be allowed in the diet for every four ounces of urine passed.

**Ingestion of Fluid.**—Another important question is how much water should be allowed in cases of Bright's disease. Dixon Mann points out that when the volume of urine excreted is ample, liquids need not be restricted, but when little urine is passed the amount of water ingested must be only so much as the kidneys can excrete. This may be determined by weighing the patient, an increase of weight showing that water is being retained in the tissues. He does not approve of the plan of giving large quantities of fluid to promote diaphoresis. Richter also holds that it is impossible to flush out the system and so eliminate toxins by large doses of water, as the fluid only accumulates in the tissues; while Kövesi and Schulz, while disagreeing with Richter on the subject of salt, agree with him in restricting the fluid ingesta. They allow one pint of liquid for every two pints of fluid excreted; it would seem, however, more scientific to adopt Dixon Mann's system of weighing the patient rather than to lay down any hard and fast rule which is unlikely to be applicable to all cases. In curious opposition to this view is that of Bacelli.[12] He insists that in the early stages of acute nephritis, after withdrawing a considerable amount of blood from a tributary of the inferior cava, large quantities of distilled water should be given to eliminate the sodium chloride, and consequently to lessen the œdema. Bacilli, however, is not likely to find many supporters, as it is very generally held that a definite tendency of the tissues to absorb water (whether or not owing to sodium chloride retention) does exist in Bright's disease, and hence no flushing-out system is practicable.

**Diaphoresis.**—Closely allied to the question of how much water should be allowed is the question as to the value of diaphoresis. Kövesi and Schulz show that 10 to 20 per cent. of the solid constituents of the urine may be removed by the skin, and it appears that in disease the skin is capable of excreting more urea than in health (Leube [13]), but these writers state that when retention of water is very great only moderate sweating should be encouraged. From the clinical standpoint Dixon Mann also supports this view, and Humphreys [14] has suggested that as damaged and devitalised cells allow more solid constituents to pass through them by dialysis, the vitality of the skin might artificially be reduced in conditions where it is desired to save the work performed by the kidneys.

**Diet.**—The question of diet has given rise to some difference of opinion in practice. As to the value of milk in acute cases there is little doubt among most clinicians, though Woods Hutchinson considers it harmful owing to the usual bacterial contaminations. This, however, is no argument against the use of a properly produced milk. In subacute and chronic cases milk should not be the sole diet. As Dixon Mann points out, it is bulky, and the amount of liquid which has to be taken is prejudicial. He advises a moderate amount of solid food, special care being taken to exclude extractives (purins) and an excess of proteid and salt. Carbohydrates and fats must make up the deficiencies in the diet. Shattock [15] advises a similar line of treatment, and states that starvation for a few days is often valuable in acute cases. In chronic interstitial forms he recommends a varied diet, restricted in quantity rather than in quality, *excess* of proteid food and alcohol being carefully avoided. Porter [16] gives an almost similar opinion as to the line of treatment to be adopted in chronic cases. He sums up the principles to be followed under three heads, gentle exercise in the open air to increase oxygenation of the tissues, warm clothing to promote continuous gentle perspiration, and a mixed diet. In a disease characterised by progressive anæmia he points out that milk is by no means a suitable food, as it is deficient in nucleo-albumin, in the molecule of which phosphorus and iron exist organically combined. He advises red meat, as the most easily digested proteid, eggs coming next, and milk third. Vegetable substances are not so digestible, but should be included in moderate amount. Leroy [17] has advised that meat should be given boiled, in order to avoid the ingestion of extractives.

**Drugs** in the treatment of nephritis are not usually considered as of very great importance; at any rate, as compared with the regulation of the diet and general mode of life of the patient. Dixon Mann speaks highly of some of the newer diuretics derived from bodies of the purin series in cases of œdema, such as caffeine, theobromine, and theocine. Theocine, which is the most powerful diuretic, increases the solid as well as the liquid constituents of the urine; the dose should not exceed six or seven grains, as it is liable to cause nausea and vomiting. When, however, the renal epithelium, on which these bodies have a specific action, is profoundly damaged, theocine may fail, and then it should be given in combination with digitalis. The double salts of theobromine and theocine with sodium, known respectively as agurine and theocine sodium acetate, are useful. Meinertz [18] states that the latter body acts as a specific in furthering chloride elimination, and Nucci [19] finds that agurine is well tolerated in large doses. It is valuable in interstitial nephritis, is more powerful and less toxic than diuretin (a double salt of theobromine with sodium salicylate), and may be given to adults in doses of fifteen grains five times in twenty-four hours. Nitroglycerin and similar substances have been given in cases of chronic Bright's disease with a view of lowering the arterial tension. Loomis [20] has carefully investigated this matter both clinically and experimentally, and his conclusions are distinctly against the use of nitroglycerin. By means of the Riva Rocci sphygmometer he found that in healthy persons nitroglycerin varied much in its action, and in subjects of chronic Bright's disease he found the blood-pressure actually rose in some cases after its administration, while in dogs the arterial effect of corresponding doses was very transient and slight, while its depressant action on the heart was the chief and most permanent effect produced. He thinks that it may be of some value in very early cases for the relief of such symptoms as headache etc., but that often some of the effect is rather psychical than physical. Where marked arteriosclerosis is present he considers that chloral hydrate is often useful. Thompson[21] agrees with Loomis in considering nitroglycerin an unreliable but safe drug even in large doses, but Le Fevre,[22] while deeming it of value in cases with high arterial tension and cardiac hypertrophy, thinks that it should be given cautiously and in small doses. He quotes two cases in which cerebral hæmorrhage followed on the administration of nitroglycerin in doses of $\frac{1}{25}$ grain.

[1] Med. Rec., July 29, 1905. [2] Ibid. [3] Ibid. [4] Scottish Med. and Surg. Jour., April, 1905. [5] Med. Rec., p. 197, 1905. [6] B. M. J., May 20, 1905. [7] Med. Rec., April 29, 1905. [8] Ibid., May 27, 1905. [9] Ibid. [10] Ibid. [11] Med. Chron., July, 1905. [12] Ibid. [13] Lancet, July 17, 1905. [14] Ibid. [15] Med. Rec., July 29, 1905. [16] Ibid., April 8, 1905. [17] Ibid., July 29, 1905. [18] Med. Chron. loc. cit. [19] Ibid. [20] Med. Record, March 18, 1905. [21] Ibid. [22] Ibid.

